# Characterisation of nocturnal arrhythmia avalanche dynamics: Insights from generalised linear model analysis

**DOI:** 10.1111/jsr.14465

**Published:** 2025-02-03

**Authors:** Sobhan Salari Shahrbabaki, Campbell Strong, Darius Chapman, Ivaylo Tonchev, Evan Jenkins, Bastien Lechat, Duc Phuc Nguyen, Murthy Mittinty, Peter Catcheside, Danny J. Eckert, Mathias Baumert, Anand N. Ganesan

**Affiliations:** ^1^ College of Medicine and Public Health Flinders University Adelaide South Australia Australia; ^2^ Department of Cardiovascular Medicine Flinders Medical Centre Adelaide South Australia Australia; ^3^ Discipline of Biomedical Engineering, School of Electrical and Mechanical Engineering The University of Adelaide Adelaide South Australia Australia

**Keywords:** generalised linear model, history modulation, nocturnal arrhythmia avalanches, point process, sleep stages, sleep‐disordered breathing

## Abstract

Nocturnal arrhythmia avalanche (NAA) episodes, characterised by transient non‐sustained cardiac arrhythmias during sleep, have been demonstrated as a predictor of adverse cardiovascular events. However, their dynamics and association with sleep architecture and events remain unclear. While generalised linear models (GLM) have captured sleep‐disordered breathing (SDB) dynamics, their application to NAA remains underexplored. This study explored whether changes in sleep architecture contribute to nocturnal arrhythmias and if the impact of sleep stages, SDB, and arousal events on these arrhythmias varies by demographic factors. We analysed 7341 ECG recordings from the multi‐ethnic study of atherosclerosis (MESA) and the sleep heart health study (SHHS) datasets. R‐R intervals were divided into 10‐min periods to detect NAA, defined as a 30% drop from baseline followed by recovery to 90% of baseline. A GLM framework was developed to characterise NAA episodes as functions of SDB, sleep arousal events, sleep stages, and prior NAA episodes. The GLM analysis revealed that NAA occurrence was 18% and 30% higher during non‐rapid eye movement (NREM) light sleep compared with deep sleep in SHHS (*p* < 0.001) and MESA (*p* < 0.001), respectively. SDB events increased the NAA risk in 34% of participants, and arousals in 29%. In SHHS, the impact of SDB on NAA was 5% greater in men (*p* = 0.018), while the arousal effects were more pronounced in those over 75, highlighting the role of demographic factors in modulating arrhythmia risk. These findings demonstrate the utility of the GLM framework in modelling the dynamics of nocturnal arrhythmias and their associations with sleep disruptions and architecture.

## 1 INTRODUCTION

A wide range of arrhythmias occur in sleep, from benign sinus bradycardia to hypoxia‐associated ventricular ectopy (Gula et al., 2004). These rhythm disturbances often mirror fluctuations in autonomic tone across different sleep stages. While arrhythmias during sleep can directly endanger patients, leading to conditions such as sudden cardiac death in congestive heart failure (Ludka et al., 2011; Verrier & Josephson, 2009), those observed during overnight monitoring might also indicate underlying issues such as obstructive sleep apnea (OSA) (Di Fusco et al., 2020; Rossi et al., 2013). These arrhythmias may present with symptoms such as palpitations or secondary angina, or they may be discovered incidentally during Holter or inpatient monitoring (Gula et al., 2004).

Arrhythmias during overnight sleep, particularly atrial fibrillation (AF), have been a subject of growing interest due to their impact on cardiovascular health. Emerging evidence suggests that sleep‐disordered breathing (SDB) plays a crucial role in developing these arrhythmias (Gami et al., 2013; Mehra et al., 2022; Monahan et al., 2009). In fact, SDB is independently associated with nocturnal arrhythmias with a higher risk for any cardiac arrhythmia with a higher severity of SDB after adjustment for a range of confounders including age, bodyweight, gender, and cardiovascular (CV) disease (Selim et al., 2016). Repetitive hypoxia and increased nocturnal sympathetic activity during SDB significantly raise the risk of complex arrhythmias, with individuals experiencing severe cases having a two‐ to four‐fold higher likelihood, even after adjusting for potential confounders (Mehra et al., 2006). Understanding the relationships between SDB and nocturnal cardiac arrhythmias is important to guide potential mutual mechanisms, future research directions, and improved patient outcomes through more mechanistically targeted therapeutic interventions. Furthermore, nocturnal arrhythmias have been shown to predict adverse CV outcomes (Azarbarzin et al., 2021; Shahrbabaki et al., 2024). The NAA episodes vary in duration and frequency, encompassing both single‐beat and multi‐beat arrhythmic episodes, and their distribution generally follows power‐law behaviour. Our recent findings demonstrate a robust association between the power‐law properties of nocturnal arrhythmia avalanches (NAA) and CV outcomes, offering novel insights for risk stratification and targeted therapeutic interventions (Shahrbabaki et al., 2024).

Sleep‐disordered breathing is a highly dynamic time‐varying process influenced by various intrinsic and extrinsic parameters such as sleep architecture, body position, sleep stage, time of night effects, and fluid retention (Chen et al., 2022; Thomas et al., 2020; White et al., 2015). The development of stochastic models of event timing dynamics has previously been utilised in neuroscience (Sarmashghi et al., 2021) and sleep studies (Chen et al., 2022; Lo et al., 2004; Shahrbabaki & Baumert, 2023; Thomas et al., 2020). Chen et al. introduced a statistical model framework grounded in point process theory. This framework effectively characterises the relative impacts of sleep stage, body position, and prior respiratory event activity on the moment‐to‐moment rate of respiratory event occurrence (Chen et al., 2022). In addition, point process theory was applied in another study to characterise moment‐by‐moment dynamics of periodic limb movements (Shahrbabaki & Baumert, 2023).

Similarly, NAA is a dynamic process that involves complex interactions between various factors, including circadian rhythms (Hayter et al., 2021), sleep architecture (Gula et al., 2004) through the impact of sleep stages on arrhythmogenesis (Verrier & Josephson, 2009), respiratory events, and autonomic nervous system (ANS) responses (de Zambotti & Baker, 2021; Gula et al., 2004).

In this study, we aimed to leverage a point process and generalised linear model (GLM) analysis framework to explore the dynamics of nocturnal arrhythmia avalanches, their relationship with sleep stages, and the occurrence of SDB and sleep arousal events. We hypothesised that changes in sleep architecture, upper airway obstruction, and sleep disruptions from arousals may contribute to the development of NAAs and influence their magnitude and duration. Furthermore, we hypothesised that the impact of sleep stages, adjacent SDB and sleep arousal events, captured by GLM, can vary by participants' demographic and anthropometric characteristics.

## 2 METHODS

This study aimed to uncover patterns and factors influencing arrhythmia during sleep using a point process GLM framework, ideal for analysing time‐to‐event data and understanding the dynamics of nocturnal arrhythmia. This approach uses local Bernoulli or Poisson models to analyse the relationship between events (Chen & Brown, 2013). A point process GLM models the expected rate of arrhythmia occurrences over time, accounting for factors such as sleep stages, sleep‐disordered breathing, arousals, and prior arrhythmic episodes. It operates as a Poisson GLM to capture the instantaneous rate of NAAs as a function of predictor variables (Chen & Brown, 2013). This approach accounts for event history, allowing us to assess how past NAA episodes influence future ones. Below, we outline the specific methodology employed in our study.

### 2.1 Data sources and sleep scorings

We analysed polysomnography data from two sleep datasets, the sleep heart health study (SHHS) and the multiethnic study of atherosclerosis (MESA), obtained from the National Sleep Research Resource (https://sleepdata.org/datasets). More details about datasets are provided in Section S1.

Trained sleep technicians blinded to all data utilised established guidelines (Berry et al., 2012; Chen et al., 2015; Iber et al., 2007) to score sleep stages, SDB, and sleep arousal events. In this study, sleep stages were labelled as wake, rapid‐eye‐movement sleep (REM), light sleep (stages 1 and 2 non rapid eye‐movement sleep [NREM]), and deep sleep (Stage 3 NREM). The SDB events included any hypopnea, obstructive sleep apnea (OSA), and central sleep apnea (CSA) episodes. In both cohorts, most individuals had OSA rather than CSA, highlighting the predominant type of SDB observed.

### 2.2 Nocturnal arrhythmia avalanche detection

We analysed PSG‐derived ECG data to detect R‐R time intervals using the Pan‐Tompkins algorithm (Pan & Tompkins, 1985). The quality of ECG recordings was assessed using signal quality indices (Zhao & Zhang, 2018). Nocturnal arrhythmia avalanches were identified by detecting drops in R‐R intervals exceeding 30% below the baseline, defined as the mean R‐R interval within a 10‐min period. An episode was considered terminated when the R‐R interval recovered to 90% of the baseline value (Section S2) (Shahrbabaki et al., 2024).

### 2.3 Development of generalised linear model

**FIGURE 1:**
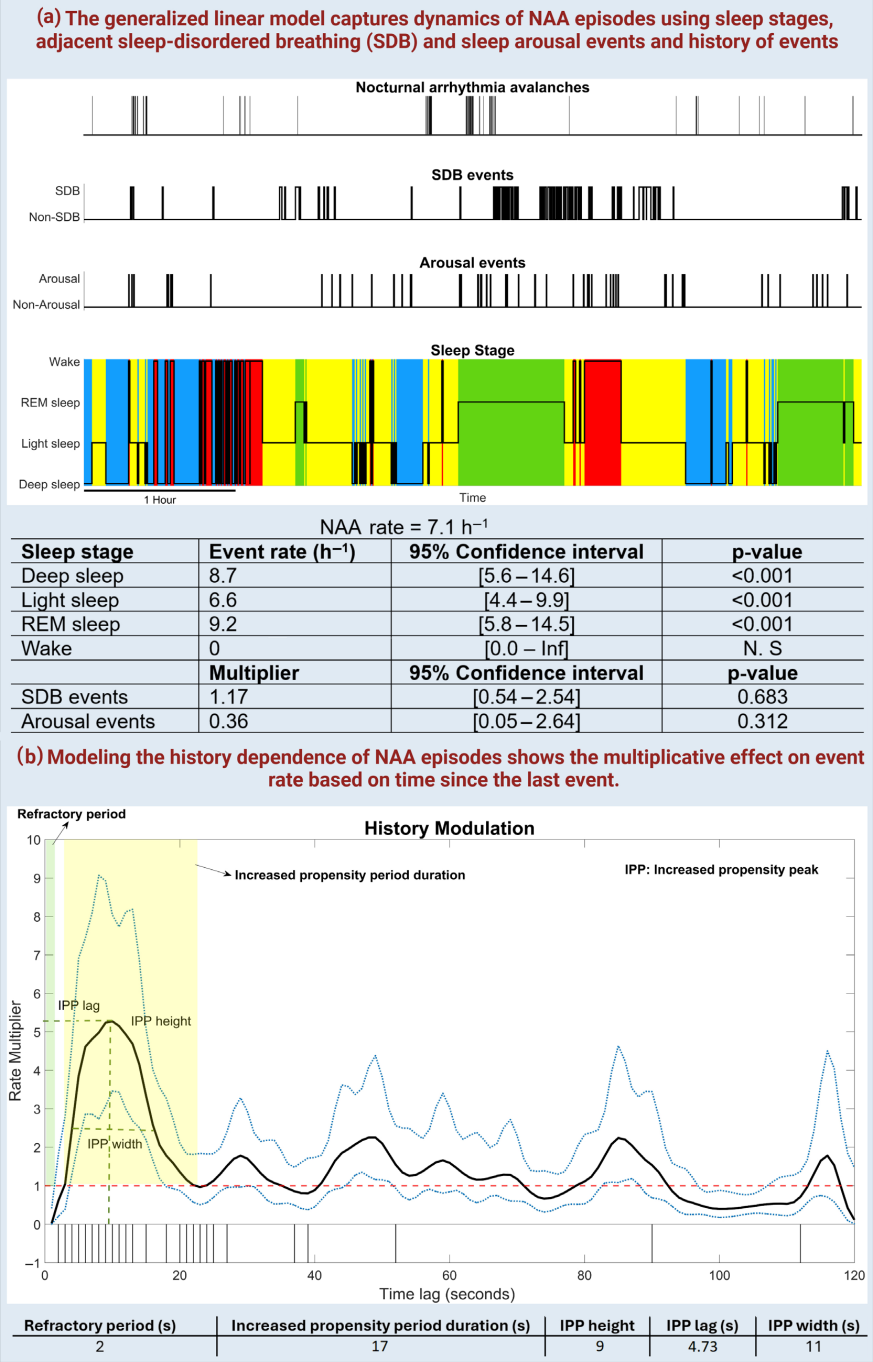
A graphical overview employing a generalised linear model (GLM) to capture the dynamics of nocturnal arrhythmia avalanches in a random participant. (a) The GLM evaluates the association of NAA occurrences with sleep stages, adjacent sleep‐disordered breathing (SDB), and sleep arousal episodes, as well as the history of NAA episodes. SDB and arousal multipliers represent the impact of respiratory and arousal events on NAA occurrence, while sleep stage event rates show the probability of NAA occurrence in each sleep stage for this participant. (b) The history modulation plot (black curve) illustrates the multiplicative effect on event rate based on the time elapsed since the last observed event, with 95% confidence intervals shown by blue dashed lines. The aligned inter‐event intervals (vertical lines at the bottom) reflect the structure of the history modulation curve, which the GLM model fits. Key features extracted from the history modulation curve include the refractory period (light green), increased propensity period duration (light yellow), and increased propensity peak height, lag, and width.

Building on the study by Chen et al. (2022), we employed a point process framework to develop a statistical model for characterising the dynamics of nocturnal arrhythmias (Figure S1). This model captures the temporal patterns and estimates the moment‐by‐moment NAA event rate, focussing specifically on the contributions of pre‐NAA factors, including sleep stage (as a continuous state), adjacent SDB, and sleep arousal events, and the timing of previous NAA episodes, to the likelihood of a current NAA occurrence (Figure 1). Additionally, the generalised linear model quantifies the association between sleep architecture, sleep disruptions, and the probability of a NAA episode occurring at any given moment as follows (Figure S1):

(1) $$\log\left(\lambda \left(t|H_{t}\right)\right)\;=\;\overset{\mathrm{SDB}}{\overbrace{\beta_{\mathrm{SDB}}I_{\mathrm{SDB}}\left(t\right)}+}\overset{\text{Arousal}}{\overbrace{\beta_{\mathrm{Ar}}I_{\mathrm{Ar}}\left(t\right)}+}\overset{\text{Sleep Stages}}{\overbrace{\sum_{s\in \mathrm{SS}}\beta_{s}I_{s}\left(t,s\right)}+}\overset{\text{History}}{\overbrace{\sum_{k=1}^{K}h_{k}g_{k}\left(H_{t}\right)}}$$

where *λ*(*t|H*_*t*_) is the instantaneous rate at time *t*, given *H*_*t*_, the history of NAA episodes up to, but not including time *t*. Furthermore, SS represents the set of sleep stages including deep sleep, light sleep, REM, and wake. The *β*_SDB_ and *β*_Ar_ are multipliers for SDB and sleep arousal, respectively, indicating and denoting the impact of adjacent SDB and arousal events on NAA, whereas *β*_*s*_ represents the model parameters reflected with the impact of sleep stages on NAA. The indicator functions *I*_*SDB*_(*t*), *I*_*Ar*_(*t*), and *I*_*s*_(*t*, *s*) are computed as follows:

(2) $$I_{\mathrm{SDB}}\left(t\right)=\left\{\begin{array}{cc} \;1 & \;\text{if}\;\mathrm{any}\;\mathrm{SDB}\;\mathrm{at}\;t\\ \;0 & \;\text{if}\;\mathrm{no}\;\mathrm{SDB}\;\mathrm{at}\;t \end{array}\right.$$

(3) $$I_{\mathrm{Ar}}\left(t\right)=\left\{\begin{array}{cc} \;1 & \;\text{if}\;\mathrm{any}\;\text{sleep arousal}\;\mathrm{at}\;t\\ \;0 & \;\text{if}\;\mathrm{no}\;\text{sleep arousal}\;\mathrm{at}\;t \end{array}\right.$$

and

(4) $$I_{s}\left(t,s\right)=\left\{\begin{array}{l} \;1\;\text{if sleep stage}=s\;\mathrm{at}\;t\\ \;0\;\text{otherwise} \end{array}\right.$$

In addition, $g_{k}\left(H_{t}\right)$ represents a collection of cardinal spline basis functions that smooth the influence of the event history $H_{t}$. The parameter K denotes the number of basis functions used to capture history dependence, while $h_{k}$ contains the model parameters that describe the past NAA activity within the cardinal spline basis and determine the likelihood of event patterns. Cardinal splines, which utilise third‐order polynomials to interpolate between points, have found application in modelling neural spike patterns in electrophysiology data (Chen et al., 2022; Sarmashghi et al., 2021). The model outputs instantaneous arrhythmic episode rates, quantifying the effect of sleep stages, adjacent SDB, and arousal events on nocturnal arrhythmias.

To establish an initial NAA history, we used the first 120 s of sleep, chosen for its lack of prior event history, providing an unbiased starting point for the model's history dependence. We then fitted the history dependence using a cardinal spline basis function with a tension parameter of 0.5. The endpoints (0 and 120 s) capture the short‐term history of NAA dynamics, with knots at −5 and 130 s to compute derivatives at the boundaries. The five inner knots were empirically placed between the 10th percentile of inter‐event intervals and 80 s to capture event history variability, with an additional knot at 100 s to account for longer‐term dependencies while preventing overfitting.

To explore the impact of the history of NAAs, we calculated a 95% upper confidence bound and lower confidence bound for the modulation curve at each time lag using model parameters. Subsequently, we defined and derived the following model parameters (Figure 1b):

**Refractory period:** The initial lag time at which the upper confidence bound of the modulation curve is below 1. It represents the period immediately following an arrhythmia episode during which the probability of another episode occurring is low during the heart's recovery phase.
**Increased propensity (IP) period duration:** The time span from when the lower confidence bound first equals or exceeds 1 until it drops back to 1 or below, indicating how long the heart remains at an elevated risk for another NAA following an initial episode.
**Increased propensity peak height:** The maximum value of the history modulation, indicating the highest probability of an arrhythmia occurring in response to a previous episode. A higher peak height may suggest a greater influence of a past NAA on the likelihood of subsequent NAAs.
**Increased propensity peak lag:** The specific time lag corresponding to the peak height. It indicates the most critical time point after an initial NAA when the heart is most susceptible to another episode.
**Increased propensity peak width:** The half peak bandwidth of the history modulation that reflects the time span of elevated arrhythmia risk. A wider peak indicates a prolonged risk period, while a narrower peak may suggest a shorter, more acute risk period.

These parameters can provide a comprehensive picture of how past NAAs influence the timing and likelihood of future episodes, helping to identify critical periods of vulnerability and potential targets for intervention.

### 2.4 Statistical analysis

We used *χ* ^2^ test to compute the effect of sleep stages, sleep arousal, and SDB events. To assess the performance of the GLM in capturing nocturnal arrhythmia dynamics, we employed the Kolmogorov–Smirnov (KS) test. The KS test is a non‐parametric test used to compare the predicted cumulative distribution function (CDF) with the observed CDF. Specifically, it measures the maximum absolute difference between these two distributions, which is known as KS statistics. The KS test was considered to have been passed for a subject if the KS statistic was below a predetermined threshold, indicating that the model predictions were sufficiently close to the observed outcomes. Notably, the critical threshold (*D*‐value) for the KS statistic varies with the sample size for each subject, leading to differences in how passing and failing the test are determined across subjects. This variability reflects the subject‐specific nature of the KS test thresholds. Furthermore, we utilised CDF to evaluate the distribution and impact of SDB and arousal multipliers across participants. A two‐sample Student's *t*‐test was used to compare model‐extracted features in men and women. ANOVA analysis was used to evaluate whether the age‐related and bodyweight‐related differences in extracted features were significant. Permutation tests with global bounds were performed to compare history curves across all groups. ANCOVA analysis was performed to evaluate the association between age and NAA dynamics, adjusted for cardiovascular conditions.

## 3 RESULTS

### 3.1 Study population

Only participants with manually scored SDB events, sleep arousal events, and detected NAA episodes during their entire sleep period were included. Participants lacking any of these criteria were excluded. As summarised in Table S1, a total of 5465 of 5793 participants (94.3%) were eligible in the SHHS (2596 men, age: 63.1 ± 11.2 years, body mass index (BMI): 28.1 ± 5.1 kg/m^2^), while 1876 of 2037 participants (92.1%) were eligible in the MESA (868 men, age: 69.5 ± 9.1 years, BMI: 28.6 ± 5.5 kg/m^2^).

### 3.2 Generalised linear model captures nocturnal arrhythmia dynamics

Figure 1 provides an overview of the GLM model characterising NAA dynamics for a random participant. The average NAA rate was approximately 7.1 events per hour, with the highest NAA rate occurring during REM sleep at 9.2 events per hour. This was followed by deep NREM sleep at 8.7 events per hour and light NREM sleep at 6.6 events per hour. No NAA episodes were detected during wakefulness in this participant. The figure also summarises statistics extracted from history modulation, indicating that the durations of the two significant regions, the refractory period and the increased propensity period, were 2 and 17 s, respectively.

The GLM exhibited robust predictive performance in capturing nocturnal arrhythmia avalanche dynamics across the study population. The KS test for the GLM passed in 65% of cases in the SHHS dataset and 53% in the MESA dataset, demonstrating predictive accuracy for the majority of participants (Figure S2). The average KS statistic was 0.176 in SHHS and 0.296 in MESA, reflecting the model's ability to characterise NAA dynamics reliably, despite some variability in predictive performance across datasets.

### 3.3 History modulation of nocturnal arrhythmia avalanches reveals significant variations across participant characteristics

**FIGURE 2:**
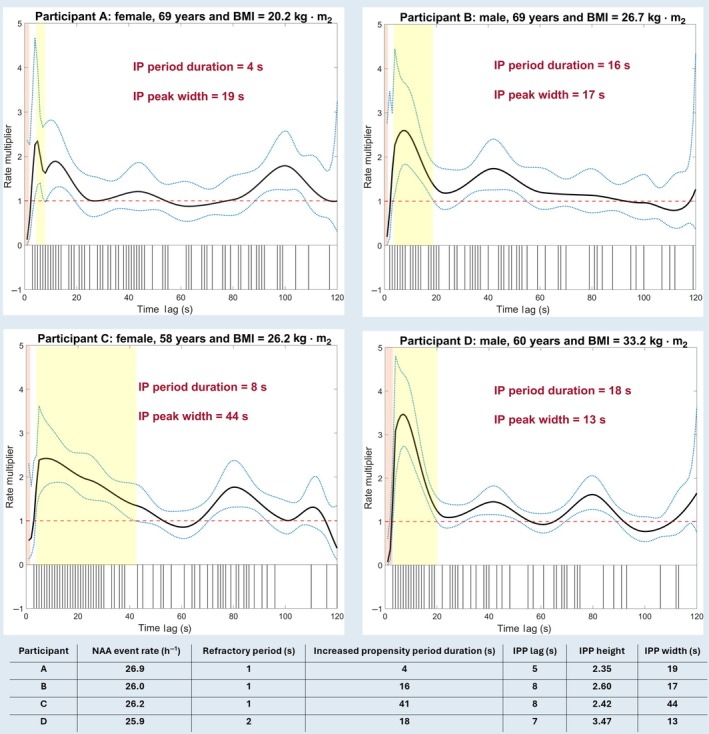
Comparison of modulation characteristics in nocturnal arrhythmia avalanches (NAA) for four participants with different demographic and anthropometric characteristics but similar event rates. The extracted features from their modulation history are summarised in the table below.

Figure 2 compares the history of NAA modulation across four participants with a similar NAA rate ≈26 events per hour but with different characteristics. Participants A and C were women of different ages and bodyweights. In Participant C, who was younger but had a greater BMI, the IP period duration was twice as long as in Participant A. Similarly, the IP peak width was 2.3 times longer in Participant A compared with Participant C. Conversely, the IP period duration in Participant A was a quarter of that in Participant B, a man of the same age but with a higher BMI, despite their almost similar IP peak widths (Participant A: 19 s vs Participant B: 17 s). Participant D, categorised as obese (BMI > 30 kg/m^2^), had the lowest IP width compared with the other three participants.

### 3.4 Impact of sleep events and sleep stages on nocturnal arrhythmia occurrence

**FIGURE 3:**
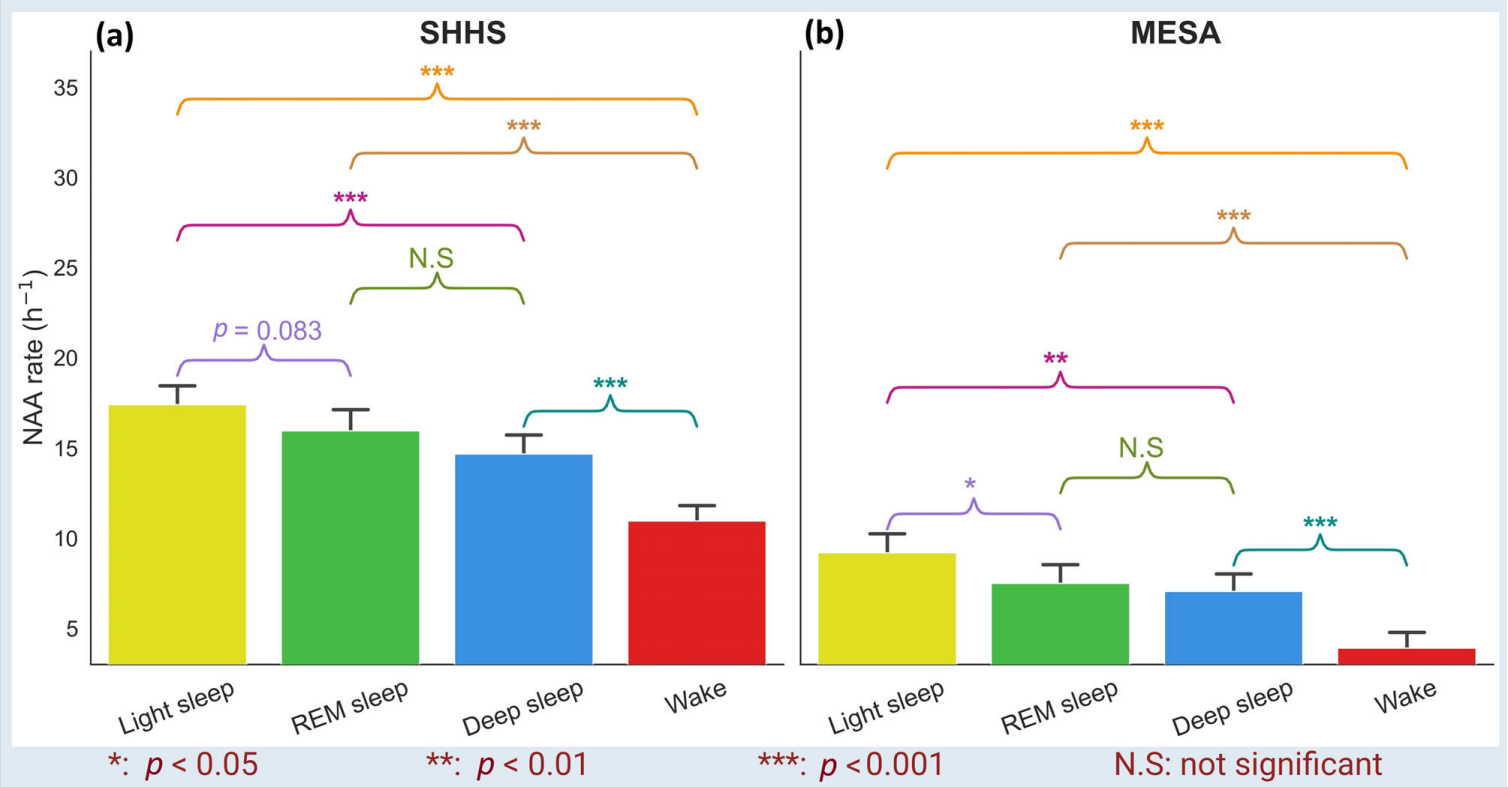
Bar‐plots compare the probability of occurrence of nocturnal arrhythmia avalanches (NAA) in different sleep stages across all participants in (a) Sleep heart health study (SHHS) and (b) Multi‐ethnic study of atherosclerosis (MESA) datasets.

The rate of NAAs during sleep was significantly higher compared with wakefulness (Figure 3, SHHS: Sleep: 16.0 ± 41.0 vs Wake: 11.0 ± 32.4 events per hour, *p* < 0.001; MESA: Sleep: 7.9 ± 20.4 vs Wake: 3.9 ± 15.9 events per hour, *p* < 0.001). Within sleep stages, the NAA rate in light NREM sleep was nearly 18% (*p* < 0.001) and 30% (*p* < 0.001) higher than in deep sleep in the SHHS and MESA datasets, respectively. In the MESA dataset, the probability of NAAs during light NREM sleep was 22% (*p* = 0.016) greater than during REM sleep, while in the SHHS dataset, the difference was marginally not significant (*p* = 0.083).

**FIGURE 4:**
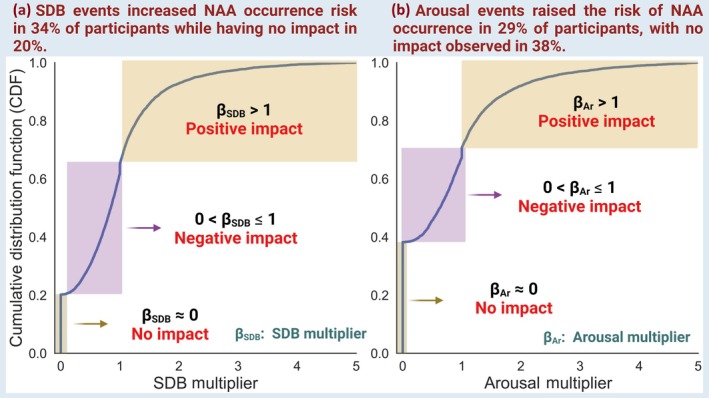
Cumulative distribution of function of sleep‐disordered breathing (SDB) and arousal multipliers assessed the impact of sleep‐disordered breathing and sleep arousal events on the occurrence of nocturnal arrhythmia avalanches.

Figure 4 presents the cumulative distribution function of SDB and arousal multipliers (*β*_SDB_ and *β*_Ar_) across all participants. The SDB multiplier or β_SDB_ exhibited values greater than 1 in more than 34% of participants, which indicates the contribution of adjacent SDB in increasing the risk of occurrence of nocturnal arrhythmia avalanches. On the other hand, in 46% of participants, the *β*_SDB_ fell between 0 and 1, indicating a negative impact of SDB on the likelihood of NAA occurrence. In the remaining participants, the *β*_SDB_ ≈ 0 suggests no impact of adjacent sleep‐disordered breathing events on NAA. Similarly, no arousal impact was observed on NAA occurrence in 38% of participants. In 29% of participants, arousal events were associated with an increased risk of NAA. For the remaining individuals, the arousal multiplier (0 < *β*_Ar_ ≤1) indicated that arousal events may cause a decrease in NAA rate (Figure 4b).

### 3.5 Demographic factors influencing nocturnal arrhythmia dynamics

**FIGURE 5:**
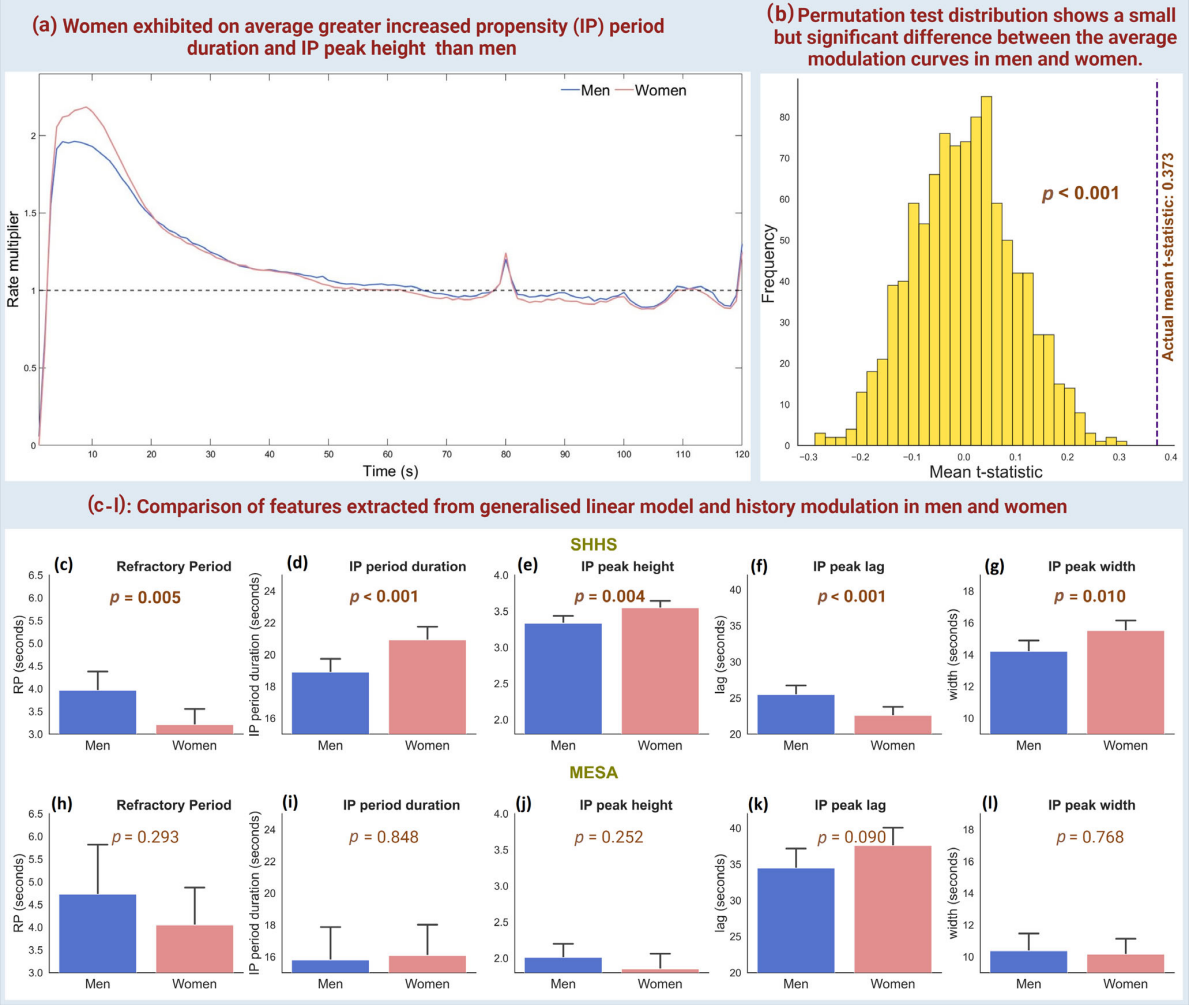
Comparison of nocturnal arrhythmia avalanches (NAA) modulation dynamics between men and women. (a) Mean history modulation curves of NAA in men versus women. (b) Permutation test distribution for modulation curve differences between men and women; Mean *t*‐statistic compared with the actual mean *t*‐statistic (0.373). (c–l) Bar plots compare features extracted from a generalised linear model and history modulation in men and women of (top) sleep heart health study (SHHS) and (bottom) multi‐ethnic study of atherosclerosis (MESA) datasets. Features include refractory period, increased propensity (IP) period duration, IP peak height, lag, and width. The *p*‐values represent *t*‐test results where significant values are denoted by bold.

**FIGURE 6:**
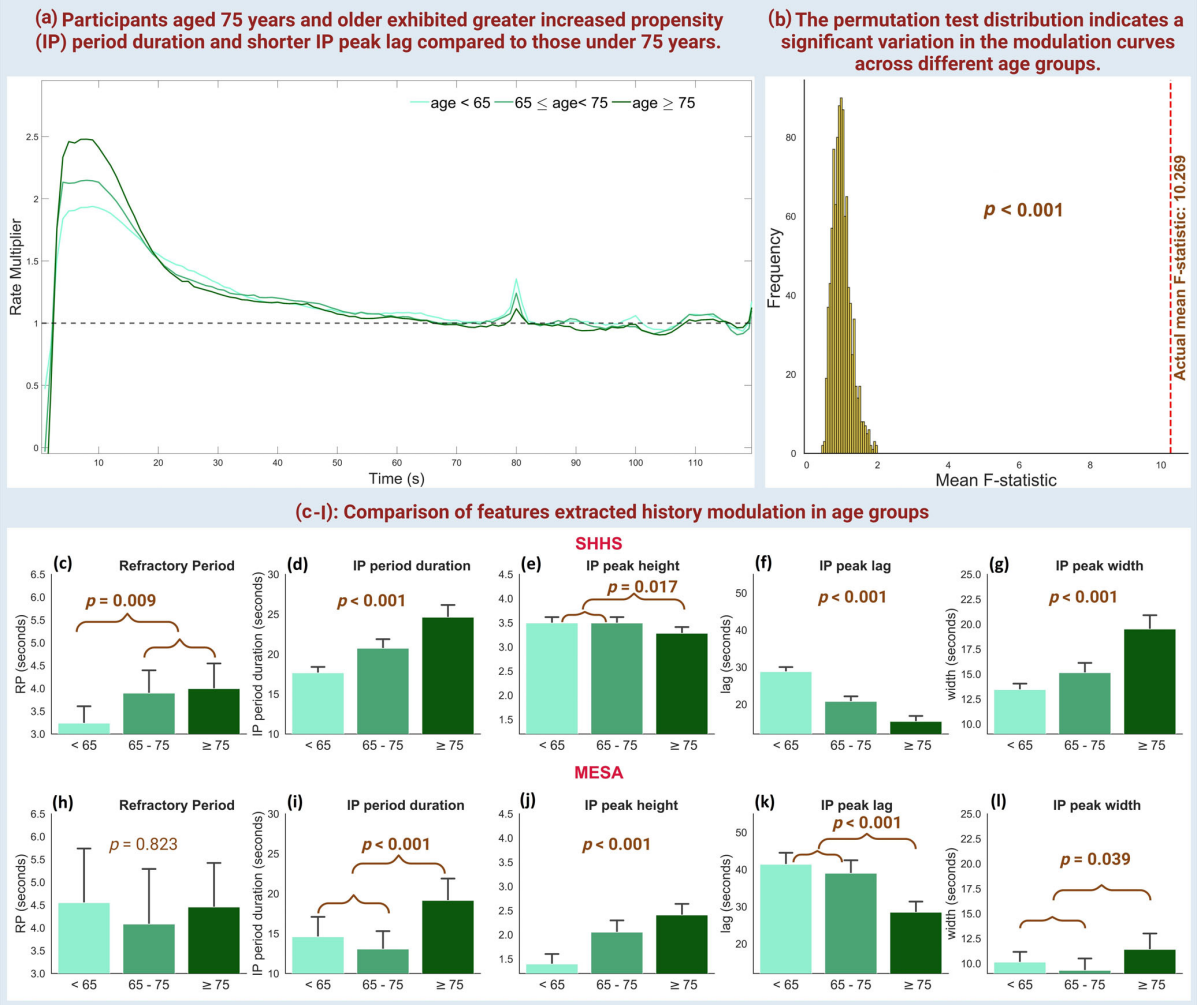
Comparison of nocturnal arrhythmia avalanches (NAA) modulation dynamics based on age (age < 65, 65 ≤ age < 75, and age ≥ 75 years). (a) Mean history modulation curves of NAA in different age groups. (b) Permutation test distribution for modulation curve differences between age groups; Mean F‐statistic compared with the actual mean F‐statistic (10.269). (c–l) Bar plots comparing features extracted from a generalised linear model and history modulation in age groups in (top) sleep heart health study (SHHS) and (bottom) multi‐ethnic study of atherosclerosis (MESA). Features include refractory period, increased propensity (IP) period duration, IP peak height, lag, and width. The *p*‐values represent ANOVA analysis results where significant values are denoted by bold.

As shown in Figure 5a, b, the mean history modulation differed significantly between women and men (*p* < 0.001), though the difference was small (mean *t*‐statistic: 0.373). Figure 5 also compares history modulation markers between men and women across both datasets. In the SHHS dataset, men had a longer refractory period compared with women (Figure 5c: men, 3.96 ± 10.29 vs women, 3.21 ± 8.94; *p* = 0.005). Conversely, the increased propensity period duration was approximately 10% longer in women (Figure 5d; *p* < 0.001). Additionally, the IP peak height and width were greater in women, by 6.2% (Figure 5e; *p* = 0.004) and 9.2% (Figure 5g; *p* = 0.010), respectively, although the IP lag was 13% longer in men (Figure 6f; *p* < 0.001). No significant associations were found between history modulation features and participant sex (Figure 5h–l). As shown in Figure S3, the probability of NAA occurrence in men was significantly greater than in women in both datasets, regardless of sleep architecture. The impact of adjacent SDBs on nocturnal arrhythmia was also greater in men than in women by approximately 5% in the SHHS (Figure S3d; *p* = 0.018), but not in the MESA (Figure S3i, *p* = 0.970).

A visual comparison of the mean history modulation curves in the three groups by age (younger than 65 years, between 65 and 75 years, and older than 75 years) shows that individuals older than 75 had the highest values, followed by younger participants (Figure 6a). The permutation test distribution indicates significant variation in the modulation curve across different age groups (Figure 6b; *p* < 0.001; Actual mean F‐statistic: 10.269). The IP period duration in participants aged ≥75 was longer by 30% (Figure 6d, *p* < 0.001) and 39% (Figure 6i, *p* < 0.001) in the SHHS and MESA, respectively. Conversely, the IP peak lag in participants older than 75 years was 69% shorter in the SHHS (Figure 6f, *p* < 0.001) and 41% shorter in the MESA dataset (Figure 6k, *p* < 0.001) compared with those younger than 75 years. Figure S4 shows a significant association between age and the probability of NAA occurrence, with older participants experiencing higher NAA rates across all sleep stages. The impact of SDB events was more pronounced in those aged ≥75 compared with those younger than 75, with increases of 7% in SHHS (Figure S4d, *p* = 0.017) and 15% in MESA (Figure S4i, *p* = 0.029). Additionally, the sleep arousal multiplier was associated with age in the SHHS dataset (Figure S4e, *p* < 0.001), while a similar association in the MESA dataset was not significant (Figure S4j, *p* = 0.083).

A comparison of history modulation features, along with the impact of sleep stage‐related rates, SDB, and arousal events across different BMI groups are presented in the Section S3, Figures S5 and S6. In summary, dependencies between history features and NREM rates with bodyweight were observed only in the SHHS dataset and not in the MESA dataset.

### 3.6 Impact of cardiovascular disease on NAA dynamics

To further explore the impact of cardiovascular history on NAA dynamics, we separated the results based on the presence or absence of atrial fibrillation and hypertension. As detailed in Section S4, Tables S2 and S3, participants with atrial fibrillation exhibited significantly higher NAA rates during light and REM sleep, along with longer increased propensity period durations and shorter IP peak lags, while hypertensive participants showed higher NAA rates, longer IP period durations and shorter IP peak lags compared with normotensive individuals.

The association between age and NAA dynamics remained significant after adjusting for the history of atrial fibrillation and hypertension (Section S5 and Tables S4).

## 4 DISCUSSION

This study was the first to utilise point process theory and generalised linear model framework to capture the dynamics of nocturnal arrhythmia and to investigate their association with sleep stages, sleep disordered breathing, and sleep arousal episodes and the history of previous arrhythmias. Our main findings indicate that: (i) The GLM framework demonstrated effectiveness in capturing NAA dynamics, as reflected by its consistent results and robust performance across two large, independent datasets. This reliability reinforces the validity of NAA impacts and suggests that the findings may be generalisable to broader populations. (ii) Significant variability in NAA modulation among participants, influenced by factors such as sex, age, bodyweight, and cardiovascular history, highlighting the complex interplay of physiological and demographic variables in sleep‐related cardiorespiratory dynamics.

Sleep represents a dynamic and temporally evolving process (Cesari et al., 2021; Prerau et al., 2014). Consequently, nocturnal arrhythmias, akin to other sleep‐related phenomena, manifest as dynamic and time‐varying occurrences influenced by various factors. Understanding the interplay between sleep phenotype and transient alterations in cardiac activity may effectively improve the management of SDB and cardiovascular disorders linked to adverse outcomes.

A greater severity of SDB independently increases the risk of nocturnal arrhythmias (Selim et al., 2016). This study aimed to quantify the impact of SDB events using the GLM framework, revealing the complex interplay between SDB severity, gender, and nocturnal arrhythmias. Our findings show that SDB events increase the NAA rates in almost a third of participants, with a stronger effect in men, consistent with the higher prevalence of atrial fibrillation and flutter, the most common arrhythmias, in men (Ehdaie et al., 2018). In addition, we introduced methodologies beyond the conventional AHI, incorporating physiological responses to SDB events and NAA dynamics. This approach helps to address problems with the AHI as a marker of OSA severity, which neglects temporal and severity characteristics and the consequences of respiratory events (Azarbarzin et al., 2024). By capturing the heterogeneity of SDB, our model provides a more nuanced understanding of its cardiovascular implications. Analysing different event types—such as hypopneas, central vs obstructive apneas, and desaturations—could further enhance our insights into their individual contributions to arrhythmogenic risk. This additional analysis might rank the arrhythmogenic potential of each event type, providing a more comprehensive assessment of their impact on cardiovascular health. Furthermore, a prospective study that includes a direct comparison between AHI and NAA dynamics in arrhythmia risk assessment and long‐term cardiovascular event prediction would help to quantify the benefits of the GLM model.

Sleep arousal and its associated ventricular repolarisation lability have been established to predict cardiovascular mortality (Shahrbabaki et al., 2021; Shahrbabaki et al., 2023). In our study, we observed a significant impact of sleep arousal on arrhythmias in almost 30% of participants. This influence was particularly notable among participants aged 75 years and older, indicating age as a contributing factor in the association between sleep arousal and arrhythmia. Our findings also align with previous studies (Bonnet & Arand, 1997; Brandenberger et al., 2005) which showed that arousal‐induced heart rate increases can disrupt heart rate variability, highlighting the cardiovascular implications of sleep disturbances.

The probability of occurrence of NAAs in sleep stages was comparably greater than in wake. The increased NAA rates during sleep align with previous studies demonstrating associations between autonomic changes, nocturnal hypoxaemia, and arrhythmias (Blanchard et al., 2021). Similar to arousal propensity, which changes markedly with sleep depth, each sleep stage has different autonomic influences over cardiac rhythm and haemodynamics (Gula et al., 2004; Penzel et al., 2003). Furthermore, sleep apnea‐related arrhythmias, including atrial fibrillation and ventricular arrhythmias, may be more likely during sleep due to nocturnal hypoxaemia and altered autonomic control (Gami et al., 2004; Mehra et al., 2006). The autonomic nervous system is profoundly affected by the sleep–wake cycle, with its activity fluctuating throughout the night in response to transitions between sleep stages (de Zambotti & Baker, 2021; Mander et al., 2017). Moreover, ageing plays a key role in nocturnal ANS dysfunction, which is associated with the development of cardiovascular disease and may impact atrial fibrillation susceptibility (Fajemiroye et al., 2018). Consistent with these findings, our study also demonstrated a significant association between the participants' age and the probability of nocturnal arrhythmia occurrence across all sleep stages. While we identified that cardiovascular disease history (atrial fibrillation and hypertension) impacts NAA dynamics, we acknowledge that the physiological mechanisms underlying these changes remain to be fully elucidated. The observed NAA dynamics could be attributed to altered autonomic regulation, where sympathetic activation during arousals and parasympathetic withdrawal might play a critical role. Further research is needed to examine the causal relationship between arousals, sympathetic–parasympathetic balance, and NAA. Future studies correlating NAA dynamics with autonomic markers such as heart rate variability could provide deeper insights into the physiological mechanisms driving these arrhythmias.

The historical modulation introduced novel metrics offering insights into arrhythmia dynamics. Specifically, the increased propensity duration indicates the heightened risk period for subsequent arrhythmias following an initial episode, with variations based on participants' sex, age, and body weight. The increased propensity peak lag reflects when the heart is most vulnerable after an arrhythmia, showing a reverse association with age. Furthermore, wider peaks observed in elderly participants indicate prolonged elevated arrhythmia risk in this group. These findings align with age being a risk factor for both SDB and atrial fibrillation, highlighting the influence of demographic factors on temporal aspects of arrhythmia susceptibility. Hence, demographic parameters play a crucial role in modulating the history and likelihood of arrhythmia, highlighting the importance of considering these factors in clinical assessments and interventions.

The observed disparities between SHHS and MESA datasets in NAA dynamics likely stem from differences in participant demographics and clinical profiles. MESA participants, being older and having a slightly higher BMI, may exhibit distinct physiological responses to sleep disruptions, resulting in lower NAA rates compared with SHHS. Despite these disparities, both datasets consistently highlight the critical role of sleep architecture, SDB, and arousals in influencing NAA dynamics, emphasising the robustness of these findings across diverse populations.

Statistical modelling provides well‐established tools for quantifying uncertainty and identifying predictable patterns in dynamic data (Chen et al., 2022). Consequently, point process models provide a robust framework for improving arrhythmia management, enabling targeted interventions such as medications, electrical stimulation, or lifestyle changes during high‐risk periods.

Nocturnal arrhythmia avalanches have emerged as a novel marker of adverse CV outcomes (Shahrbabaki et al., 2024). This study introduced innovative approaches to explore the dynamic mechanisms underlying NAA and to assess the influence of sleep architecture and disruptions on their occurrence. By investigating these factors, we aimed to enhance understanding of the role of sleep in NAA risk, potentially guiding targeted interventions to reduce CV risk in patients with disturbed sleep patterns.

Furthermore, previous studies indicated that individualised sleep interventions such as continuous positive airway pressure (CPAP) therapy, commonly used to treat SDB, may help to decrease the occurrence and recurrence of arrhythmias in affected patients (Padeletti et al., 2014). Understanding arrhythmia dynamics before and after CPAP can optimise therapeutic outcomes and minimise overall nocturnal arrhythmic episodes. Future research in clinical settings should explore potential interventions based on demographic characteristics and SDB severity.

## 5 LIMITATIONS

Our study's findings are based on single‐night in‐home overnight polysomnography recordings, and highlight potential night‐to‐night variations in arrhythmia dynamics (Linz et al., 2018). A greater understanding of this variability and its influence on critical sleep parameters and cardiovascular outcomes is needed to further extend our findings (Lechat et al., 2023; Scott et al., 2023). The retrospective design limits causal inference, and the study populations' characteristics constrain generalisability, though consistent findings across two large cohorts suggest robustness. The predominantly white, middle‐aged to older cohorts further limit applicability. Our study design precluded the investigation of nocturnal arrhythmia avalanches during waking hours and the GLM performance was limited in participants with low NAA episodes. While the mean KS statistic summarises model performance, it may not fully capture individual variability, highlighting the importance of considering the distribution of KS statistics across participants. Future studies should refine the model by incorporating additional data sources or extending the monitoring period. Exploring alternative modelling approaches and integrating more diverse cohorts could also enhance the generalisability and robustness of the findings. The differences between datasets may reflect methodological factors, including variations in scoring protocols, equipment sensitivity, and statistical power due to sample size. Future studies with standardised protocols and larger, diverse populations are required to validate these findings.

## 6 CONCLUSION

The generalised linear model framework effectively captures the dynamics of nocturnal arrhythmias and their associations with sleep disruptions and architecture. Among the different sleep stages, non‐rapid eye movement light sleep exhibits the highest likelihood of arrhythmia occurrence, followed by REM sleep and deep non‐REM sleep. Additionally, SDB and arousal were associated with the risk of NAA occurrence in almost one‐third of participants. Furthermore, demographic factors play a crucial role in modulating arrhythmia history and likelihood, emphasising the importance of considering them in clinical assessments and interventions.

## AUTHOR CONTRIBUTIONS

**Sobhan Salari Shahrbabaki:** Conceptualization; investigation; methodology; writing – review and editing; writing – original draft; visualization; software; resources; formal analysis; data curation; project administration. **Campbell Strong:** Visualization; writing – review and editing. **Darius Chapman:** Writing – review and editing. **Ivaylo Tonchev:** Writing – review and editing; validation. **Evan Jenkins:** Writing – review and editing. **Bastien Lechat:** Writing – review and editing. **Duc Phuc Nguyen:** Writing – review and editing. **Murthy Mittinty:** Writing – review and editing; validation. **Peter Catcheside:** Writing – review and editing. **Danny J. Eckert:** Writing – review and editing. **Mathias Baumert:** Data curation; writing – review and editing; validation. **Anand N. Ganesan:** Supervision; writing – review and editing; funding acquisition; validation.

## FUNDING INFORMATION

The study was funded by the National Health and Medical Research Council Ideas Grant 2010522.

## CONFLICT OF INTEREST STATEMENT

The authors declare no conflicts of interest.

## Supporting information


**Data S1** Supporting Information.

## Data Availability

The data that support the findings of this study are openly available in National Sleep Research Resource at https://sleepdata.org/.
