# Supplementary material for: Characterisation of nocturnal arrhythmia avalanche dynamics: Insights from generalised linear model analysis
Source: J Sleep Res. 2025 Feb 3;34(6):e14465. doi: 10.1111/jsr.14465 (PMC12592826; doi:10.1111/jsr.14465)
Supplement: Supplementary file 1 — Data S1 Supporting Information. [file JSR-34-e14465-s001.doc]

**Online Supplementary material**

**Characterization of Nocturnal Arrhythmia Avalanche Dynamics: Insights from Generalized Linear Model Analysis**

AUTHOR LIST:

Sobhan Salari Shahrbabaki^1^, Campbell Strong^1^, Darius Chapman^1^, Ivaylo Tonchev^1,2^, Evan Jenkins^1^, Bastien Lechat^1^, Duc Phuc Nguyen^1^, Murthy Mittinty^1^, Peter Catcheside^1^, Danny J Eckert^1^, Mathias Baumert^3^, Anand N Ganesan^1,2^

Institution List:

1. College of Medicine and Public Health, Flinders University, Adelaide, Australia
2. Department of Cardiovascular Medicine, Flinders Medical Centre, Adelaide, Australia
3. Discipline of Biomedical Engineering, School of Electrical and Mechanical Engineering, University of Adelaide, Adelaide, Australia

**Section S1: Data acquisition**

In this study, we analyzed polysomnography data from two sleep datasets, the Sleep Heart Health Study (SHHS) and the Multiethnic Study of Atherosclerosis (MESA), obtained from the National Sleep Research Resource (<https://sleepdata.org/datasets>).

**The Sleep Heart Health Study (SHHS)** conducted under the leadership of the National Heart, Lung, and Blood Institute, is a prospective investigation into the correlation between obstructive sleep apnea (OSA), other sleep-disordered breathing (SDB) conditions, and cardiovascular (CV) disease risk. The study enrolled participants from established cohort studies concentrating on cardiovascular or respiratory conditions. Specifically, SHHS targeted individuals who had not undergone treatment for SDB with continuous positive airway pressure (CPAP), did not have a tracheostomy, and were not undergoing home oxygen therapy (Haas et al., 2005). Out of the initial 11,503 eligible individuals from the parent cohort studies, 6,841 participants underwent a home overnight polysomnography sleep study between November 1995 and January 1998 (Haas et al., 2005; Redline et al., 1998). We had access to PSG data from 5,793 participants.

**Multi-Ethnic Study of Atherosclerosis (MESA)** is a research project examining both clinical and subclinical factors that contribute to the progression of atherosclerosis (Bild et al., 2002; Huang et al., 2020). This study involved a total of 6814 men and women from six different locations across the United States. Institutional review board approval was obtained at each site, and all participants provided written consent. Between 2010 and 2013, 2261 participants underwent a comprehensive evaluation of their sleep patterns, which included an at-home polysomnography (PSG) session and completion of sleep-related questionnaires. Out of these participants, 2037 individuals had PSG results that met the required technical criteria.

**In-home overnight PSG and sleep scoring:** Sleep recordings were performed using an unattended, portable in-home PSG over one night at the participant's residence using Somte sleep monitoring system (Compumedics, Abbotsford, Victoria, Australia) for MESA (Chen et al., 2015), and Compumedics P-series for SHHS (Punjabi et al., 2009). Trained staff members visited the participants to attach the sensors and electrodes and conduct overnight PSG. The PSG setup included several measurements such as two central electroencephalograms, bilateral electrooculograms, bilateral chin electromyogram, a bipolar electrocardiogram, nasal-oral thermistor, nasal flow via pressure transducer and nasal cannula, abdominal and respiratory inductance plethysmography, finger pulse oximetry, bilateral leg movements monitored by piezoelectric sensors, and body position (Baumert et al., 2019; Chen et al., 2015; Punjabi et al., 2009). In the MESA sleep study, fingertip photoplethysmography recording was also available (Shahrbabaki et al., 2023). Trained sleep technicians, blinded to all other data utilized established guidelines (A. Rechtschaffen & Kales, 1968; Berry et al., 2012; Chen et al., 2015) to score sleep stages, SDB and arousal events in order to derive apnoea-hypopnea index (AHI), arousal burden (AB) (Shahrbabaki et al., 2021), oxygen desaturation index (ODI) and total time spent below 90% oxygen desaturation (T90). This standardized approach allowed for objective and quantitative evaluations of sleep architecture and sleep-disordered breathing.

### **Section 2: ECG signal processing**

In the MESA and SHHS cohorts, ECG recordings were extracted from polysomnography data stored in European Data Format (EDF) files. To ensure the quality of each segment, a 5-second window was selected throughout the recordings. Within each window, the kurtosis of ECG normal sinus (kSQI) was calculated and evaluated to identify and remove any noisy segments based on specific criteria (Zhao & Zhang, 2018):


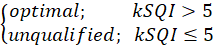
 (1);

R-R intervals, representing the time intervals between successive heartbeats, were detected and estimated using the Pan-Tompkins algorithm (Pan & Tompkins, 1985). The detected R-R intervals were then evaluated using the cSQI, defined as the ratio of the mean to the standard deviation of the RR interval distribution. This assessment was used to identify and remove low-quality ECG signals as follows (Zhao & Zhang, 2018):


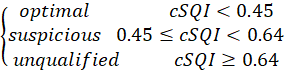
 (2);

We devised the Signal Quality Ratio (SQR) by calculating the ratio of 5-second windows in the ECG recording with optimal kSQI and cSQI to the entire recording. Participants exhibiting an SQR<60% were categorized as having inadequate ECG and were subsequently excluded from the analysis.

ECG recordings were divided into 10-minute segments with 2-second overlaps. The baseline was established for each 10-minute segment and used to detect nocturnal arrhythmia avalanche (NAA) episodes. QRS-template matching was employed to identify and exclude ventricular ectopic beats from the analysis. Additionally, all RR intervals shorter than 250 milliseconds were excluded. NAAs were identified by detecting drops in RR intervals exceeding the threshold. The termination of an episode was defined as the point when the R-R interval synchronized and returned to 90% of the baseline.(Shahrbabaki et al., 2024)

**Section 3: Bodyweight-related differences in nocturnal arrhythmia dynamics**

We investigated the association between extracted features from GLM modelling of NAA events and participants' body weight after categorizing them into three groups based on their body mass index (BMI): Normal (BMI≤25 kg/m²), overweight (25<BMI≤30 kg/m²), and obese (BMI>30 kg/m²). The permutation test revealed significant but minor differences in mean history modulation among normal, overweight, and obese individuals (**Figure S5-A** and **B**, *p*=0.002, Actual mean F-statistic: 2.149).

In the SHHS dataset, the increased propensity (IP) period duration was inversely associated with BMI, with higher BMI corresponding to shorter IP periods (**Figure S5-D**, Normal: 21.9±19.57, Overweight: 19.96±18.48, Obese: 18.31±18.07, *p*<0.001). Participants with BMI >30 kg/m² had an average IP peak lag 9% longer than those with BMI ≤30 kg/m² (*p*=0.002). Conversely, the IP peak width was 8.5% longer in obese participants compared to others (**Figure S5-G**, *p*=0.009). There was no significant association between body weight and history modulation features in the MESA dataset (**Figure S5-H** to **L**).

As shown in **Figure S6**, in the SHHS cohort, the NAA event rate in NREM light and deep sleep was higher in individuals with BMI ≤25 kg/m² compared to those classified as overweight and obese by 13% (*p*=0.058) and 21% (*p*=0.014), respectively. Neither events rate in different sleep stages nor SDB and arousal multipliers were associated to bodyweight in the MESA dataset (**Figure S6-F** to **J**).

**Section 4: Influences of cardiovascular history on NAA dynamics**

To examine the influence of participants' cardiovascular history on NAA dynamics, we compared individuals with a history of atrial fibrillation (AF) and flutter to those without AF in both datasets (**Table S2**). The NAA event rate during light sleep was significantly higher in participants with AF, being 6.7 times that of non-AF participants in SHHS (*p*<0.001) and 2.6 times in MESA (*p*=0.002). Similarly, the event rate during rapid eye movement (REM) sleep was approximately four times greater in AF participants compared to non-AF participants in SHHS (*p*<0.001) and three times greater in MESA (p=0.019). In terms of IP dynamics, AF patients exhibited a significantly longer increased propensity (IP) period duration in both cohorts (SHHS: 34.67±29.10 seconds in AF vs. 19.47±19.81 seconds in non-AF, *p*<0.001; MESA: 59.71±38.38 seconds in AF vs. 18.47±27.56 seconds in non-AF, *p*=0.002). Conversely, IP peak lag was markedly shorter in individuals with AF, being less than half that observed in non-AF participants in SHHS (*p*<0.001). Similarly, in MESA, the IP peak lag in AF participants was, on almost one-quarter that of non-AF individuals (*p*<0.001).

As shown in **Table S3**, participants with a history of hypertension had higher NAA event rates during light and REM sleep compared to normotensive individuals in both the SHHS and MESA cohorts. In SHHS, hypertensive participants had event rates of 23.18±50.70 vs. 13.43±34.81 h^-1^ in light sleep (*p*<0.001) and 21.41±49.24 vs. 12.23 ± 36.32 h^-1^ in REM sleep (p<0.001). Similarly, in MESA, hypertensive participants had event rates of 10.89 ± 23.06 vs. 7.27±17.32 h^-1^ in light sleep (*p*<0.001) and 8.92±21.77 vs. 5.92±17.49 h^-1^ in REM sleep (*p*=0.002). The IP period duration was 15% (*p*<0.001) and 25% longer (*p*=0.010) in hypertensive participants compared to those without hypertension in the SHHS and MESA cohorts, respectively. On the other hand, hypertensive participants exhibited a 23% shorter IP peak lag in SHHS (*p*<0.001) and a 20% shorter lag in MESA compared to normotensive participants.

**Section 5: Impact of age on NAA dynamics considering cardiovascular conditions**

To further investigate the association between age and the dynamics of nocturnal arrhythmia avalanche, we employed an analysis of covariance (ANCOVA), adjusting for the history of atrial fibrillation and hypertension to assess the independent impact of age (**Table S4**). The variations in the rate of NAA episodes were significantly associated with participants’ age in both datasets after adjusting for cardiovascular conditions. The deep sleep NAA rate was directly associated with age, with coefficients of 0.553 (*p*<0.001) and 0.293 (*p*<0.001) in the SHHS and MESA datasets, respectively. Similar associations between age and NAA occurrence rates in light and REM sleep were observed in the SHHS (Light sleep: coefficient = 0.067, *p*<0.001; REM sleep: coefficient = 0.656, *p*<0.001) and MESA (Light sleep: coefficient = 0.447, *p*<0.001; REM sleep: coefficient = 0.352, *p*<0.001). The impact of age on SDB and arousal multiplier was small but significant only in the SHHS dataset (β_SDB_ = 0.003, *p*=0.038; β_AR_ = 0.008, *p*<0.001), and not significant in the MESA dataset after adjusting for cardiovascular conditions.

In the history modulation statistics, the increased propensity period duration was associated with age even in the presence of CV diseases in both datasets (SHHS: coefficient = 0.357, *p*<0.001; MESA: coefficient = 0.308, *p*<0.001). The IP peak height was directly associated with age (SHHS: coefficient = 0.029, *p*<0.001; MESA: coefficient = 0.043, *p*<0.001), while the IP lag was inversely associated with age (SHHS: coefficient = -0.756, *p*<0.001; MESA: coefficient = -0.573, *p*<0.001).

**References:**

**Tables:**

**Table S1**: Characteristics of the multi-ethnic atherosclerosis study (MESA) and the sleep heart health study (SHHS) cohorts. AHI: apnea/hypopnea index

| **Variable** | **SHHS** | **MESA** |
| --- | --- | --- |
| **Number of participants (n)** | 5465 | 1876 |
| **Men (n)** | 2596 | 868 |
| **Age (years)** | 63.1±11.2 | 69.5±9.1 |
| **Age>65 years (n)** | 2950 | 686 |
| **65≤age<75 years (n)** | 1523 | 584 |
| **Age>75 years (n)** | 992 | 606 |
| **Body mass index (kg.m^-2^)** | 28.1±5.1 | 28.6±5.5 |
| **Normal (n)** | 1502 | 516 |
| **Overweight (n)** | 2286 | 702 |
| **Obese (n)** | 1677 | 658 |
| **Total sleeping time (min)** | 356.2±64.5 | 361.8±81.6 |
| **Light sleep (min)** | 222.9±57.8 | 258.8±65.8 |
| **Deep sleep (min)** | 61.3±39.9 | 36.8±34.1 |
| **REM sleep (min)** | 72.0±27.7 | 66.3±30.1 |
| **AHI (h^-1^)** | 12.3±14.1 | 23.5±15.2 |
| **Arousal burden (%)** | 5.88±3.33 | 6.34±3.60 |

**Table S2**: Dynamics of nocturnal arrhythmia in patients with a history of atrial fibrillation (AF) and/or atrial flutter compared to those without AF (No AF) in SHHS and MESA cohorts.

|  | **SHHS** | | | **MESA** | | |
| --- | --- | --- | --- | --- | --- | --- |
| **Feature** | **AF**  **(Mean ± SD)** | **No AF**  **(Mean ± SD)** | **p-value** | **AF**  **(Mean ± SD)** | **No AF**  **(Mean ± SD)** | **p-value** |
| **β_SDB_** | 1.07 ± 0.58 | 0.97 ± 0.79 | 0.184 | 0.85 ± 0.39 | 0.64 ± 0.78 | **0.049** |
| **β_AR_** | 0.93 ± 0.40 | 0.81 ± 0.89 | **0.032** | 0.77 ± 0.44 | 0.54 ± 0.80 | 0.061 |
| **Deep sleep rate (h^-1^)** | 100.18 ± 140.00 | 14.85 ± 37.05 | **<0.001** | 18.28 ± 21.15 | 6.96 ± 20.51 | 0.104 |
| **Light sleep rate (h^-1^)** | 87.40 ± 73.00 | 17.34 ± 41.92 | **<0.001** | 34.26 ± 24.72 | 8.92 ± 20.49 | **0.002** |
| **REM sleep rate (h^-1^)** | 84.14 ± 99.35 | 16.54 ± 45.60 | **<0.001** | 22.65 ± 21.46 | 7.38 ± 19.93 | **0.019** |
| **Refractory Period (s)** | 12.79 ± 22.81 | 7.36 ± 20.66 | 0.071 | 2.20 ± 1.05 | 3.89 ± 13.27 | **<0.001** |
| **IP period duration (s)** | 34.67 ± 29.10 | 19.47 ± 19.81 | **<0.001** | 59.71 ± 38.38 | 18.47 ± 27.56 | **0.002** |
| **IP peak lag (s)** | 12.73 ± 16.46 | 26.65 ± 30.75 | **<0.001** | 9.27 ± 4.43 | 36.44 ± 34.65 | **<0.001** |
| **IP peak height** | 1.98 ± 0.89 | 3.07 ± 2.31 | **<0.001** | 2.06 ± 1.50 | 1.92 ± 2.35 | 0.717 |
| **IP peak width (s)** | 29.27 ± 26.72 | 13.86 ± 17.42 | **0.008** | 23.57 ± 16.56 | 10.18 ± 14.75 | 0.095 |

β_SDB_: sleep-disorder breathing multiplier; β_AR_: sleep arousal multiplier, REM: rapid eye movement; IP: increased propensity; SD: standard deviation

**Table S3**: Dynamics of nocturnal arrhythmia avalanches in patients with a history of hypertension (hypertensive) compared to those without hypertension (normotensive) in SHHS and MESA cohorts.

|  | **SHHS** | | | **MESA** | | |
| --- | --- | --- | --- | --- | --- | --- |
| **Feature** | **Hypertensive**  **(Mean ± SD)** | **Normotensive**  **(Mean ± SD)** | **p-value** | **Hypertensive**  **(Mean ± SD)** | **Normotensive**  **(Mean ± SD)** | **p-value** |
| **β_SDB_** | 0.98 ± 0.70 | 0.96 ± 0.77 | 0.312 | 0.66 ± 0.78 | 0.61 ± 0.76 | 0.178 |
| **β_AR_** | 0.83 ± 0.85 | 0.81 ± 0.91 | 0.476 | 0.57 ± 0.77 | 0.52 ± 0.82 | 0.183 |
| **Deep sleep rate (h^-1^)** | 19.90 ± 47.85 | 11.16 ± 29.22 | **<0.001** | 7.76 ± 19.99 | 6.28 ± 20.96 | 0.156 |
| **Light sleep rate (h^-1^)** | 23.18 ± 50.70 | 13.43 ± 34.81 | **<0.001** | 10.89 ± 23.06 | 7.27 ± 17.32 | **<0.001** |
| **REM sleep rate (h^-1^)** | 21.41 ± 49.24 | 12.23 ± 36.32 | **<0.001** | 8.92 ± 21.77 | 5.92 ± 17.49 | **0.002** |
| **Refractory Period (s)** | 7.91 ± 21.19 | 6.92 ± 20.35 | 0.087 | 4.24 ± 14.17 | 3.42 ± 11.85 | 0.188 |
| **IP period duration (s)** | 21.54 ± 19.95 | 18.62 ± 18.11 | **<0.001** | 20.72 ± 29.72 | 16.60 ± 25.54 | **0.010** |
| **IP peak lag (s)** | 20.84 ± 26.62 | 26.12 ± 30.36 | **<0.001** | 33.15 ± 33.99 | 39.62 ± 34.90 | **<0.001** |
| **IP peak height** | 3.35 ± 2.15 | 3.52 ± 2.42 | **0.024** | 2.20 ± 2.36 | 1.61 ± 2.27 | **<0.001** |
| **IP peak width (s)** | 16.18 ± 18.43 | 14.09 ± 17.30 | **<0.001** | 10.75 ± 15.44 | 9.70 ± 14.08 | 0.149 |

β_SDB_: sleep-disorder breathing multiplier; β_AR_: sleep arousal multiplier, REM: rapid eye movement; IP: increased propensity; SD: standard deviation

**Table S4**: ANCOVA results evaluating the association between age and NAA dynamics, adjusted for atrial fibrillation and hypertension in the SHHS and MESA cohorts. Coefficients represent the change in NAA dynamics associated with age, with p-values indicating statistical significance.

|  | **SHHS** | | **MESA** | |
| --- | --- | --- | --- | --- |
| **Feature** | **Coefficient** | **p-value** | **Coefficient** | **p-value** |
| **β_SDB_** | 0.003 | **0.038** | 0.004 | 0.081 |
| **β_AR_** | 0.008 | **<0.001** | 0.001 | 0.506 |
| **Deep sleep rate** | 0.553 | **<0.001** | 0.293 | **<0.001** |
| **Light sleep rate** | 0.067 | **<0.001** | 0.447 | **<0.001** |
| **REM sleep rate** | 0.656 | **<0.001** | 0.352 | **<0.001** |
| **Refractory Period** | 0.069 | **0.033** | 0.005 | 0.888 |
| **IP period duration** | 0.357 | **<0.001** | 0.308 | **0.001** |
| **IP peak lag** | -0.756 | **<0.001** | -0.537 | **<0.001** |
| **IP peak height** | 0.029 | **<0.001** | 0.043 | **<0.001** |
| **IP peak width** | 0.261 | **<0.001** | 0.062 | 0.148 |

β_SDB_: sleep-disorder breathing multiplier; β_AR_: sleep arousal multiplier, REM: rapid eye movement; IP: increased propensity

**Figures:**

**
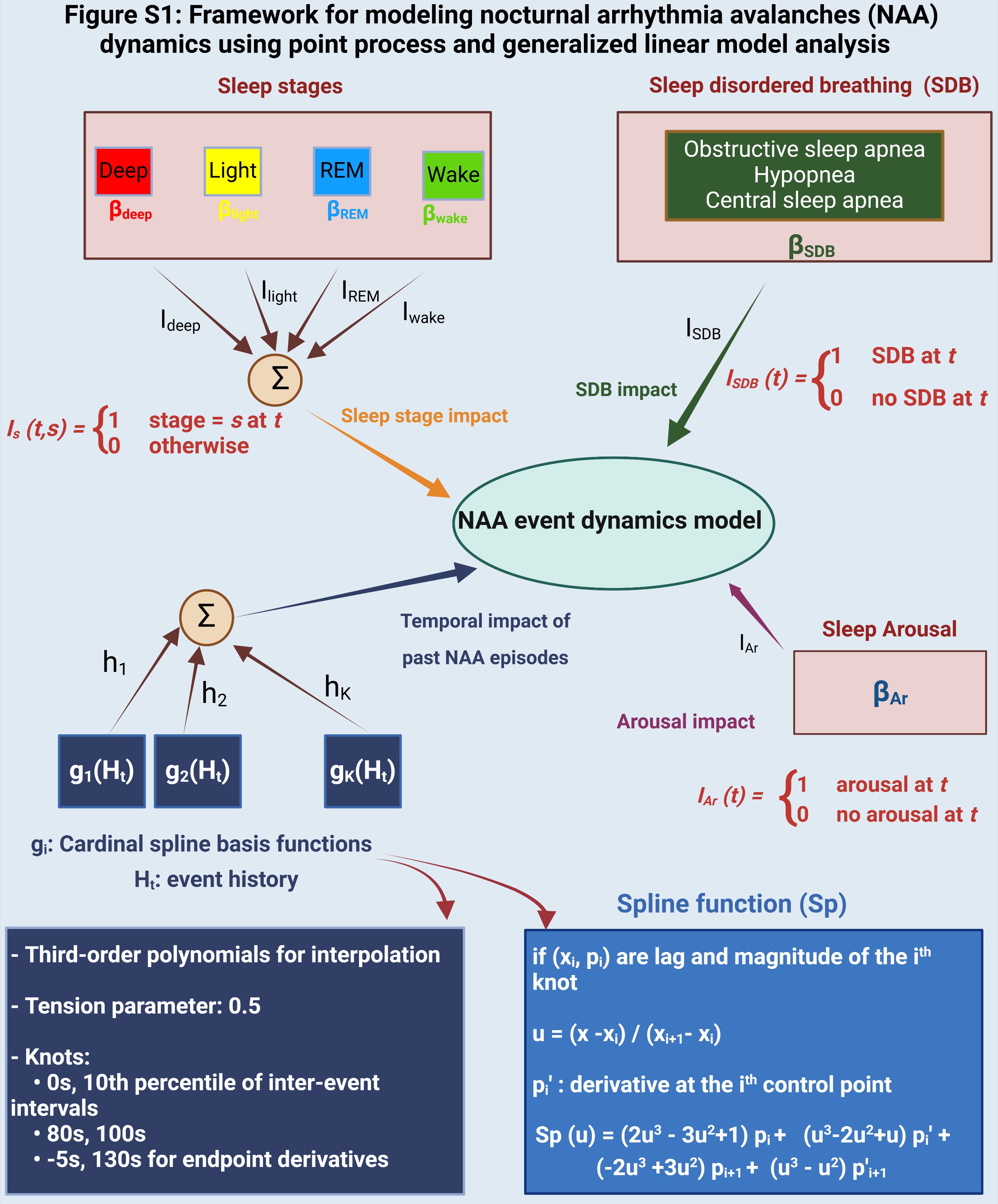
**

**Figure S1:** The flowchart illustrating the framework for modeling nocturnal arrhythmia avalanches dynamics. The central model employs a generalized linear model (GLM) with a point process framework to estimate the NAA rate. Inputs include sleep stage dynamics, adjacent sleep-disordered breathing (SDB) and sleep arousal events, and the history of past NAAs. The history is modeled using cardinal spline basis functions to capture temporal dependencies. This approach quantifies the influence of sleep architecture, disruptions, and past event patterns on the likelihood of NAAs at any given moment.

**
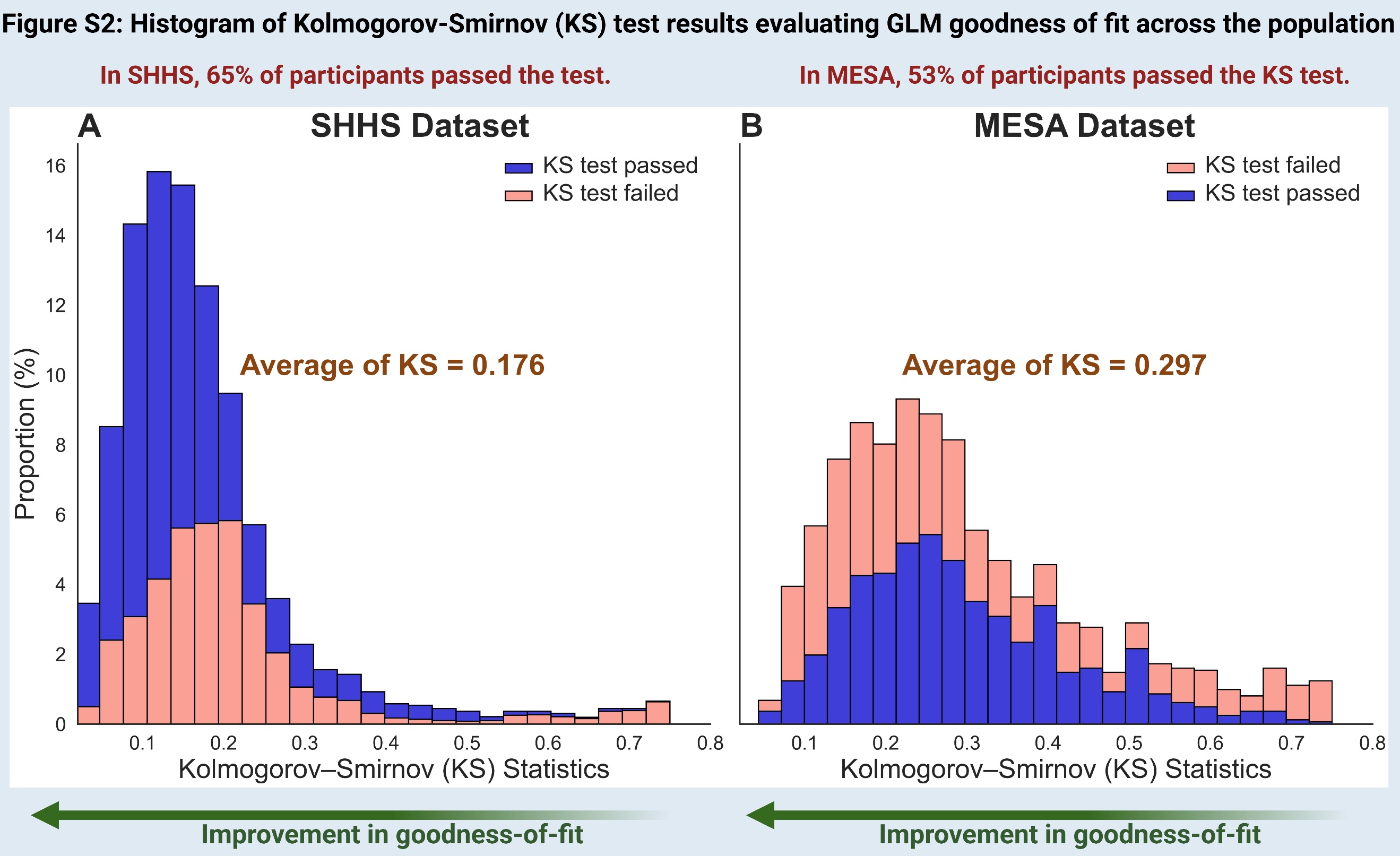
**

**Figure S2:** Kolmogorov-Smirnov (KS) statistics evaluate the goodness-of-fit of generalized linear model of nocturnal arrhythmia avalanches across the population in (A) SHHS and (B) MESA datasets.


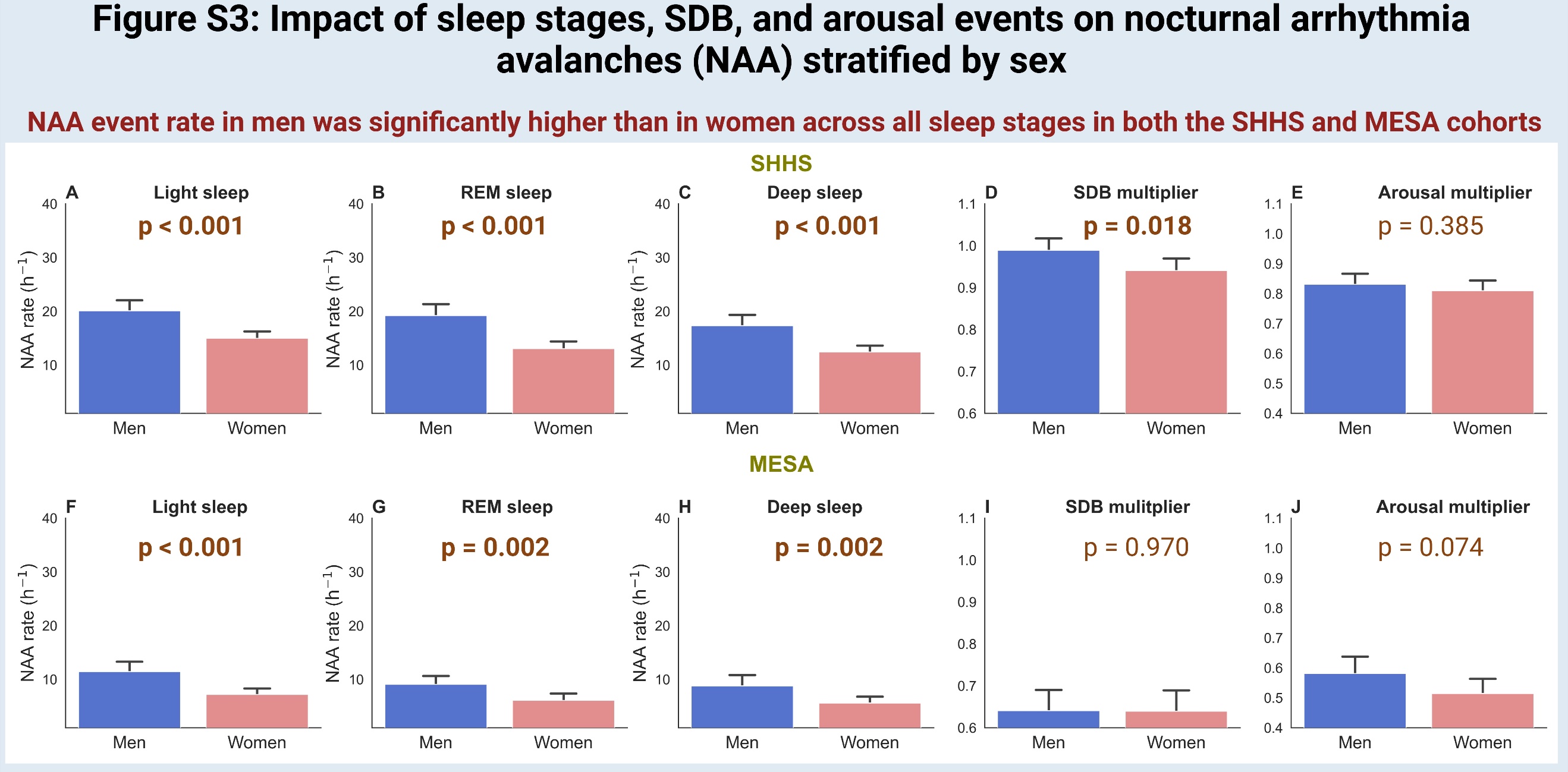


**Figure S3:** Comparison of the impact of sleep stages, sleep-ordered breathing (SDB) and sleep arousal events on occurrence of nocturnal arrhythmia avalanches (NAA) dynamics between men and women of (**top**) SHHS and (**bottom**) MESA datasets. P-values represent t-test results with significant values denoted in bold.


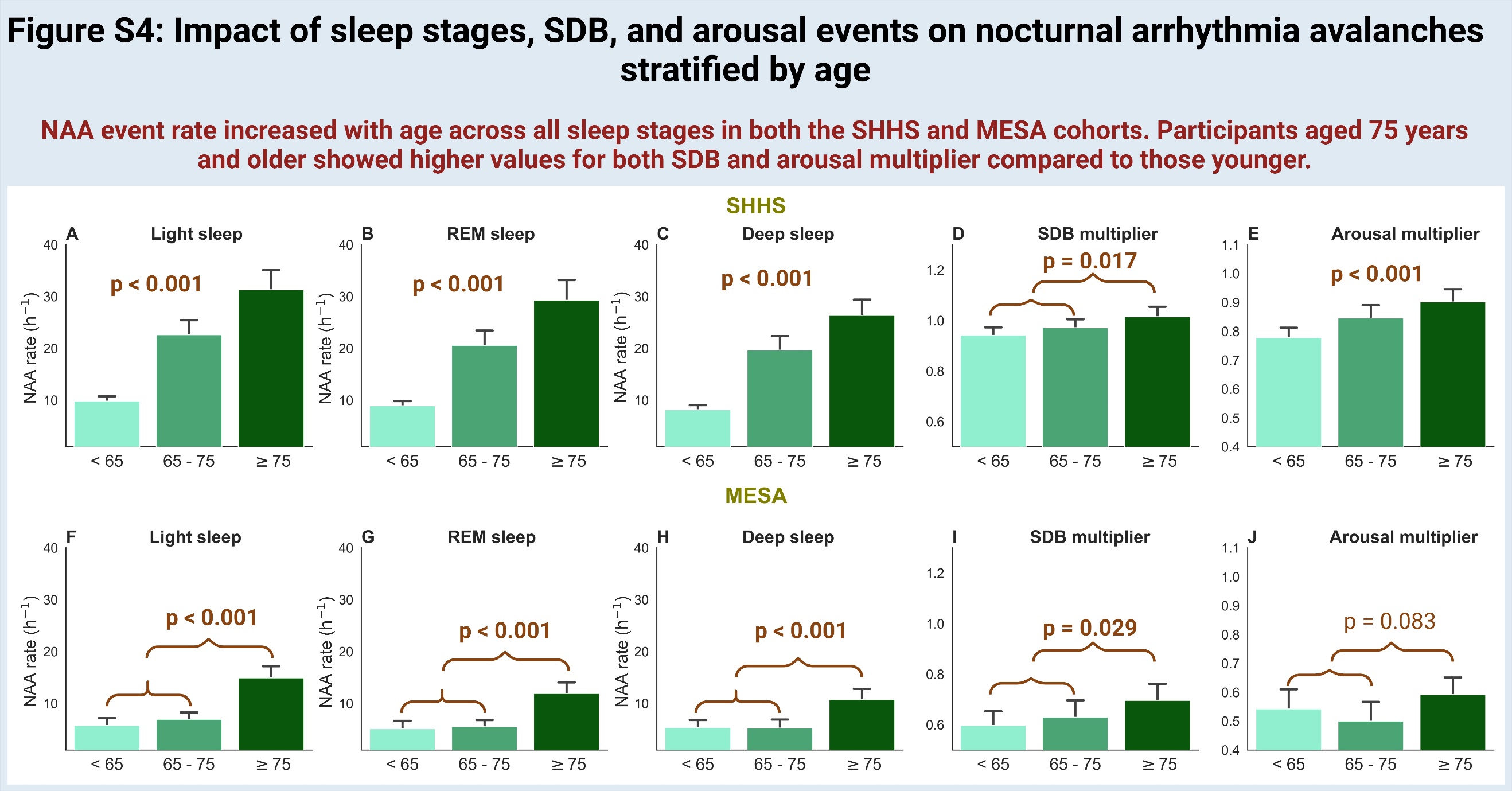


**Figure S4:** Comparison of the impact of sleep stages, sleep-ordered breathing (SDB) and sleep arousal events on occurrence of nocturnal arrhythmia avalanches (NAA) dynamics captured by a generalized linear model between different age groups (65≤age<75, and age≥ 75 years) in the (**top**) SHHS and (**bottom**) MESA datasets. P-values represent ANOVA analysis results where significant values are denoted by bold.

**
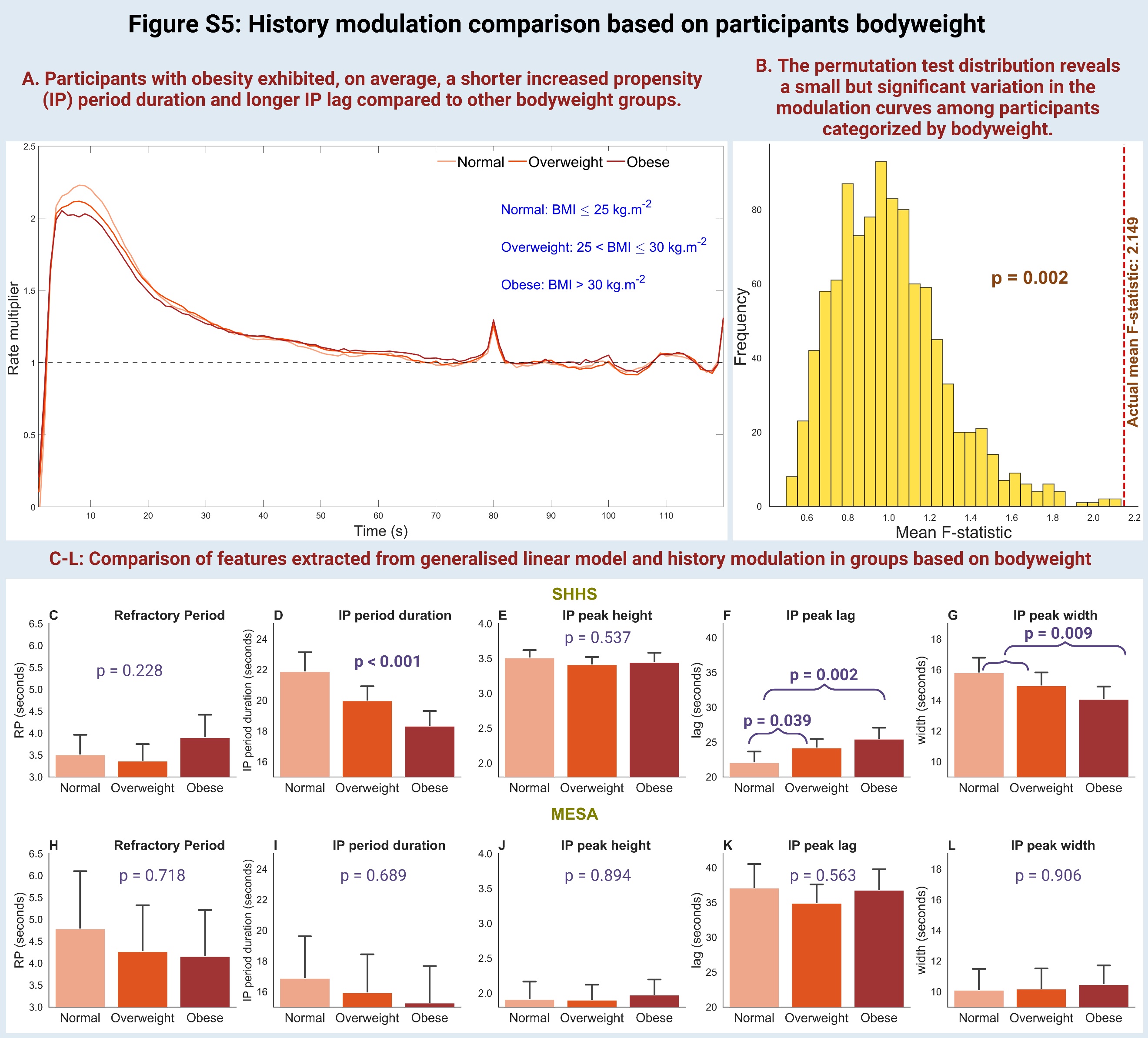
**

**Figure S5:** Comparison of nocturnal arrhythmia avalanches (NAA) modulation dynamics based on participants bodyweight. (**A**) Mean history modulation curves of NAA in different body mass index (BMI) groups (Normal: BMI≤25 kg.m^-2^; Overweight: 25<BMI≤30 kg.m^-2^; Obese: BMI>30 kg.m^-2^). (**B**) Permutation test distribution for modulation curve differences between BMI groups; Mean F-statistic compared with the actual mean F-statistic (2.149). (**C-L**) Bar plots compare features extracted from a generalized linear model and history modulation in BMI groups in (**top**) SHHS and (**bottom**) MESA. Features include refractory period, increased propensity (IP) period duration, IP peak height, lag and width P-values represent ANOVA analysis results where significant values are denoted by bold.


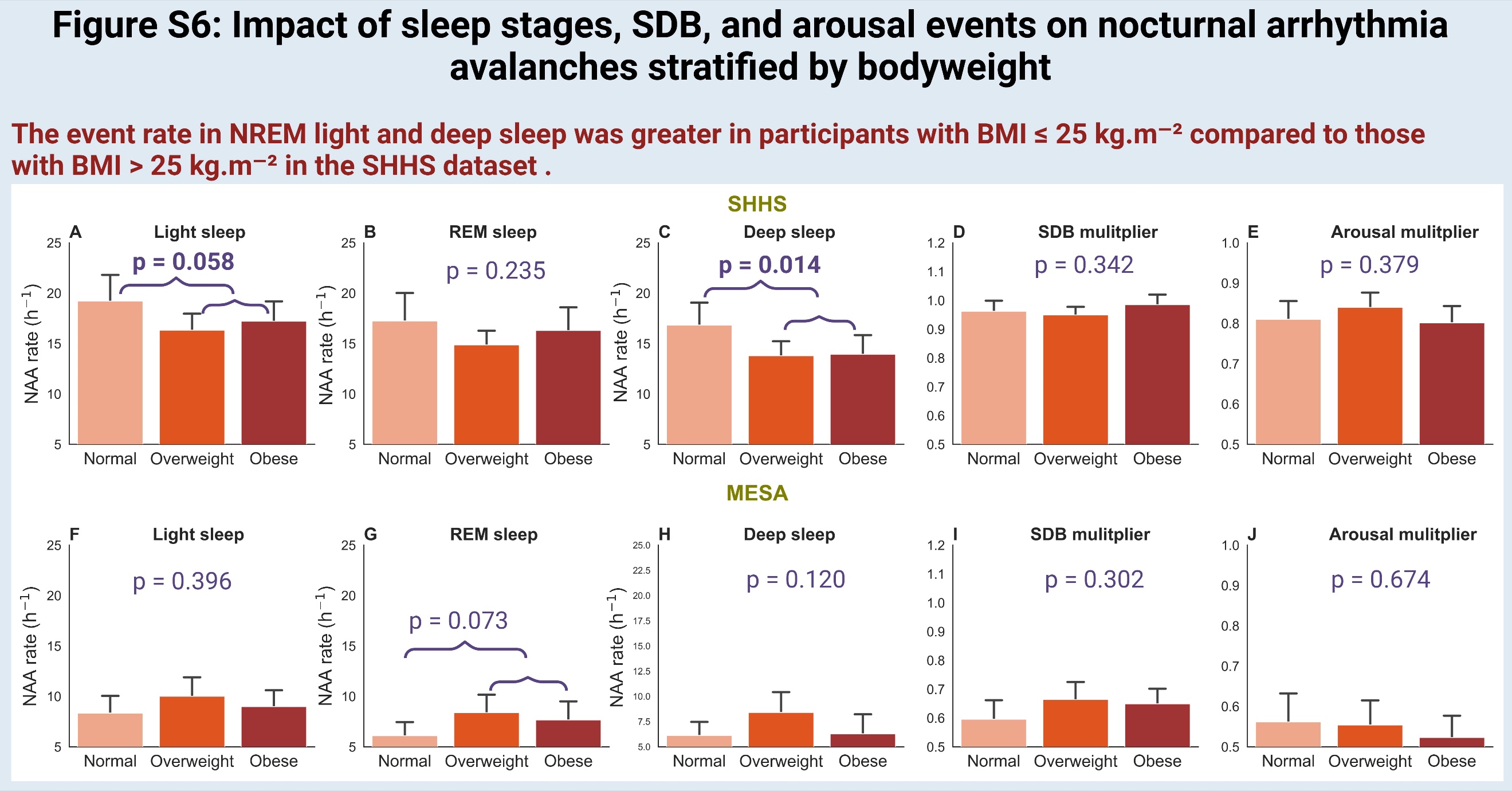


**Figure S6:** Comparison of the impact of sleep stages, sleep-ordered breathing (SDB) and sleep arousal events on occurrence of nocturnal arrhythmia avalanches (NAA) dynamics captured by a generalized linear model between different body mass index (BMI) groups (Normal: BMI≤25 kg.m^-2^; Overweight: 25<BMI≤30 kg.m^-2^; Obese: BMI>30 kg.m^-2^) in the (**top**) SHHS and (**bottom**) MESA datasets. P-values represent ANOVA analysis results where significant values are denoted by bold.
